# Transcriptional Dynamics at Brain Enhancers: from Functional Specialization to Neurodegeneration

**DOI:** 10.1007/s11910-016-0689-7

**Published:** 2016-09-14

**Authors:** Marit W. Vermunt, Menno P. Creyghton

**Affiliations:** Hubrecht Institute-KNAW and University Medical Center Utrecht, Uppsalalaan 8, 3584CT Utrecht, The Netherlands

**Keywords:** Epigenetics, Gene regulation, Enhancer, Common variation, Parkinson’s disease, α-synuclein

## Abstract

Over the last decade, the noncoding part of the genome has been shown to harbour thousands of *cis*-regulatory elements, such as enhancers, that activate well-defined gene expression programs. Driven by the development of numerous techniques, many of these elements are now identified in multiple tissues and cell types, and their characteristics as well as importance in development and disease are becoming increasingly clear. Here, we provide an overview of the insights that were gained from the analysis of noncoding gene regulatory elements in the brain and describe their potential contribution to cell type specialization, brain function and neurodegenerative disease.

## Introduction

Cell state specification is determined by the tight control of gene expression programs that arise as a result of pre-programmed developmental cascades as well as environmental stimuli [1, 2]. Transcriptional regulation is a dynamic process and is guided by transcription factors that occupy *cis*-regulatory elements (CREs) in noncoding parts of the genome. CREs are short stretches of DNA that contain recognition motifs onto which transcription factors can dock [3]. These in turn recruit cofactors that modify the local chromatin environment and that influence the assembly of a functional transcriptional apparatus at the core promoter of genes.

Our understanding of the basic principles of gene regulation and the role that transcription factors play in this process have increased substantially over the last 10 years [4]. Close to 1400 transcription factors have been identified [5] and recent advances in large-scale sequencing techniques are further expanding our insights of transcriptional regulation by allowing the identification of hundreds of thousands of CREs as well as the networks in which they operate [6]. Small sets of master regulators have emerged as central to the coordination of these transcriptional networks during development [4, 7]. Accordingly, overexpression of such factors can initiate reprogramming of fully differentiated cells towards a different developmental path [8]. Thus, while studying transcription factors and their regulatory networks has provided critical insight into the processes of cell state specification, it has also allowed us to control these states.

Numerous diseases, including most cancers, are caused by aberrant transcriptional regulation which is often the result of mutations in transcription factors or their associated cofactors [4]. Interestingly, a number of diseases have also been linked to mutations within CREs [9–11]. In addition, most single nucleotide polymorphisms (SNPs), associated with a variety of disorders, have been identified in noncoding DNA [12]. Therefore, efforts to understand the genetic and epigenetic basis of pathology have significantly shifted focus from coding sequences (i.e. genes) towards noncoding regulatory elements. This is especially the case for complex diseases such as neuropsychiatric and neurodegenerative disorders, in which much of the underlying heritability has remained elusive [13].

In the current review, we focus on gene expression control in the human brain. We summarise how large-scale identification of *cis*-regulatory DNA is starting to broaden our understanding of transcriptional programs in the brain and how this knowledge can be used to uncover gene regulatory alterations that contribute to complex brain diseases.

### Unravelling Transcriptional Programs in the Brain

For decades, scientists have been attempting to unravel the transcriptional programs that give rise to the wide variety of neuronal cell types that collectively make up the central nervous system. Several transcription factors were shown to play key roles in neurogenesis and neuronal diversification (for an extensive review see [14]). For instance, the expression of *Dlx1* (distal-less homeobox 1) and *Dlx2* counteracts the expression of *Olig1* (oligodendrocyte transcription factor 1) and *Olig2* to promote interneuron fate over oligodendrocyte specification and vice versa [15, 16]. Furthermore, upregulation of the transcription factor *Pax6* (paired box 6) induces neurogenesis through induction of *Ngn2* (neurogenin 2) [17]. Surprisingly, overexpression of *Pax6* could also induce neurogenesis in post-natal astrocytes *in vitro*, representing one of the earliest examples of lineage conversion directed by a single transcription factor [18]. *Ngn2* as well as *Ascl1* (achaete-scute homolog 1), another key factor in neurogenesis, was also shown to be able to drive neuronal cell fate specification in post-natal astrocytes suggesting that differentiation boundaries could be overcome using specific transcription factors [19]. Following these discoveries, select combinations of transcription factors were identified that, when overexpressed, were able to induce major cell state changes [8]. This included the direct conversion of fibroblasts into neuronal cell types [20], which has now been achieved through overexpression of different combinations of neuronal transcription factors, typically using *Ascl1* as a cornerstone factor (reviewed in [21]). These data demonstrate that transcription factors play a central role in determining cell state specification in the nervous system as well as in controlling the plasticity of these states.

Following the emergence of genome-scale transcriptome analyses, spatio-temporal gene expression programs in the brain are now rapidly being elucidated. Large consortia, including the Allen Institute for Brain Science and *BrainSpan*, have collected gene expression data in murine and human tissues at different developmental stages as well as in brain tissue from humans suffering from neurological disorders [22, 23]. Furthermore, co-expression analysis of these types of data has revealed a hierarchical structure of networks in which certain transcription factors present as central (hub) genes that modulate the expression of other genes [22, 24, 25]. For instance, *TBR1* (T-brain 1) and *EMX2* (empty spiracles homeobox 2) have emerged as hub regulators in the adult human brain [25] and are well-known cortical transcription factors involved in state specification of cortical progenitors and adult neurons [26]. While these analyses are starting to reveal the hierarchal structure of gene regulatory networks, a full grasp of their complexity can only be achieved when combined with intricate knowledge of the underlying CREs to which these transcription factors bind. The latter analysis has until recent years been lagging behind.

### Characteristics of *cis*-Regulatory DNA

**Fig. 1:**
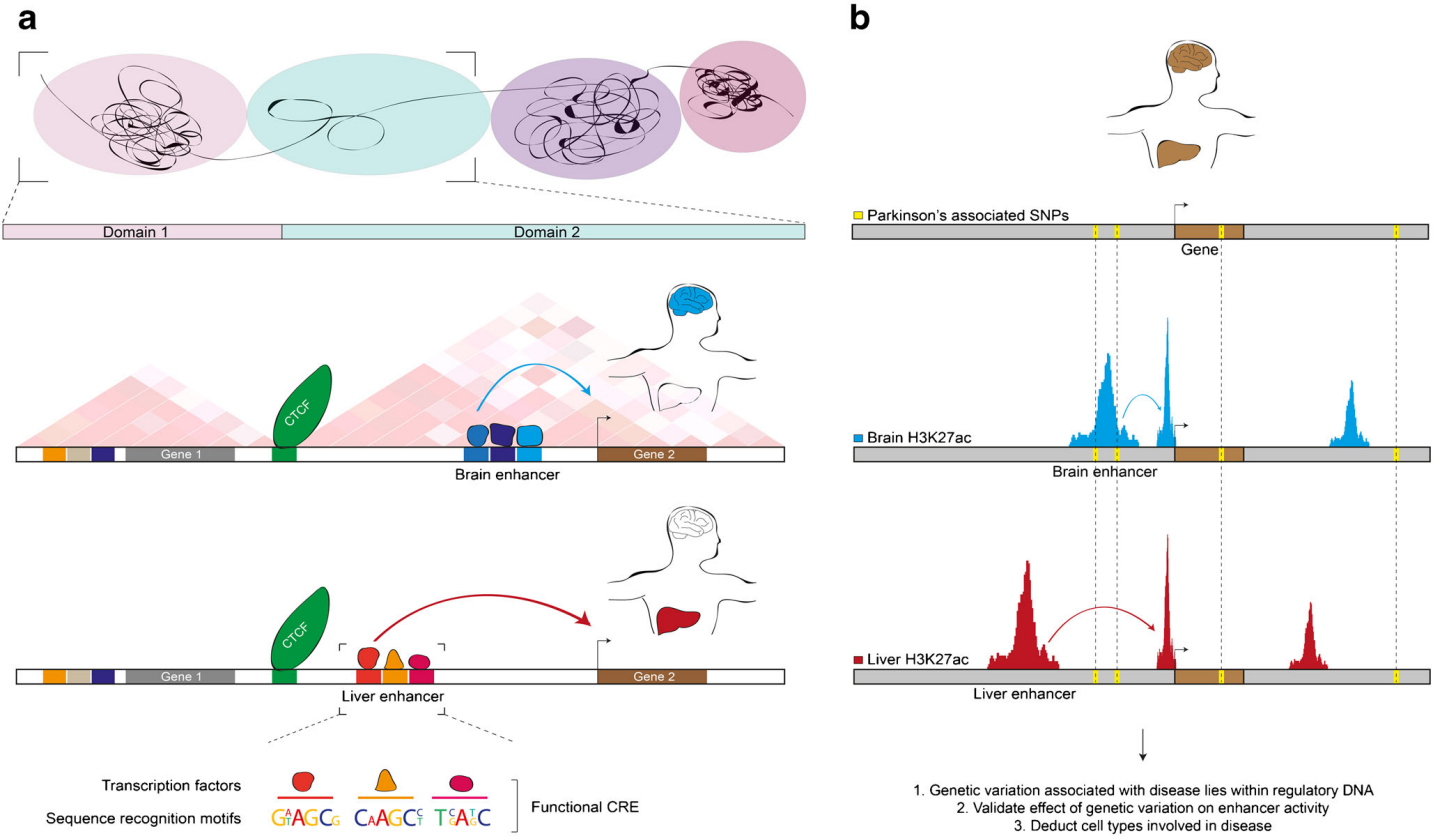
*Cis*-regulatory elements in gene expression and disease. **a**. The genome is subdivided into chromatin neighbourhoods (*upper panel*). Within domains, delineated by CTCF (*green*) and typically analyzed by chromatin conformation capture techniques (hypothetical Hi-C result shown), enhancers drive target gene expression through long-range interactions with the promoter of their target gene (two examples shown below the domains). Enhancers often regulate genes in a tissue-specific manner as depicted here for the brain (*blue*) and liver (*red*). Functional CREs comprise stretches of binding motifs that are bound by transcription factors to ultimately determine CRE activity (*bottom*). **b**. The combination of enhancer identification and GWAS studies has revealed that many single nucleotide polymorphisms (*yellow*) lie within *cis*-regulatory DNA (*blue*, H3K27ac in the brain; *red*, H3K27ac in the liver). Variation within CREs can contribute to disease susceptibility and aid in the identification of relevant cell types

CREs are short stretches of noncoding DNA, typically 200–500 base pairs in length, that contain sequence motifs that are recognized and bound by transcription factors (Fig. 1a) [27, 28]. The spacing, location and sequence content of these binding motifs can be either very relaxed or tightly determined depending on which enhancer is assayed [29]. The number of potential binding motifs within the genome for a given transcription factor typically outpaces the number of actual binding events by an order of magnitude [30]. This is primarily explained by the combinatory nature of transcription factor binding [30–32]. Typically, a hierarchical sequence of binding events will start with the docking of so-called pioneer factors that promote the accessibility of DNA to other proteins [32–34]. This will stabilize a core complex that in turn recruits other, more ubiquitously expressed, cofactors through protein-protein interactions [35]. These cofactors can further modify the local chromatin environment by adding defined epigenetic modifications to histone tails, thus creating additional docking sites for proteins and further altering local chromatin state [36]. Alternatively, they may directly influence transcription initiation at the core promoter.

The combined activity of different transcription factors bound to a single element ultimately determines its regulatory capacity as being an activator (enhancer) or repressor (silencer) of gene expression. CREs are considered part of the promoter when located next to a gene’s transcriptional start site onto which the RNA polymerase II transcription initiation complex is assembled. However, they can also be situated at large genomic distances (up to 1 million base pairs [9]) and interact with the promoter of their target gene through a process called chromatin looping [37, 38]. Several factors such as CCCTC-binding factor (CTCF), mediator and cohesin are involved in the establishment of these long-range interactions [39–41]. The genomic architecture within a cell’s nucleus is further shaped by CTCF boundaries, called insulators, into neighbourhoods in which genes and their CREs are isolated (Fig. 1a) [41–43]. Target gene regulation is often restricted to these domains and dependent on the combined activity of CREs [40]. Genes can also be regulated by multiple enhancers in a modular fashion meaning that separate enhancers support gene expression in a particular anatomical structure or cell type (Fig. 1a) [29, 32]. For instance, different neural enhancers independently regulate the expression of proopiomelanocortin across several neuroanatomical regions [44]. Therefore, sets of transcription factors operate through a number of CREs to orchestrate the activation and repression of genes into well-defined transcriptional programs.

### Genome-Wide Annotation of CREs

Early discovery of CREs relied on the careful analysis of a handful of single gene regions [45]. One of the most well-studied examples is the beta-globin locus [46]. Similar efforts have led to the identification of regulatory DNA that acts on the *Pax6* gene in the lens. Later, it became clear that *Pax6* is under the control of multiple enhancers to strictly regulate its expression in the eye, brain and pancreas [47]. This underscored the function of enhancers as modular regulators of gene expression in a cell type-specific manner.

Based on the fact that gene function is typically conserved across species, the assumption that gene regulation would be equally conserved led to the identification of several thousands of predicted enhancer sequences across species using comparative genomics [48, 49]. Typically, half of these highly conserved elements contained measurable enhancer activity when tested in transgenic animals using reporter assays and a significant portion supported expression in the developing nervous system [49, 50]. However, several enhancers that were found by analysing specific genes of interest displayed little evidence of sequence conservation, with some showing no sequence conservation despite being functionally conserved [51, 52]. This raised the question on how much regulatory information was still missing.

The realization that *cis*-regulatory DNA contains specific epigenetic footprints [53, 54], combined with the emergence of large-scale sequencing techniques to measure them genome-wide [53], has significantly propelled our understanding of the regulatory networks that dictate gene expression. For instance, CREs typically reside in open chromatin and are generally characterized by low nucleosome density in combination with defined histone variants or histone modifications [36]. The accessibility of CREs can be exploited to chart their location at a genome-wide level using a variety of large-scale assays [55–57]. While most of the identified regions are likely enhancers, open chromatin also contains insulators bound by CTCF [58], repressors such as regions occupied by REST (RE1 silencing transcription factor) [59] and potential other regulatory elements [60].

Different types of CREs are associated with distinct histone signatures and transcription factors [36, 61], the location of which can be measured by chromatin immunoprecipitation (ChIP) followed by sequencing. Promoters are mainly marked by histone 3 lysine 4 mono-methylation (H3K4me1) and H3K4me3, while H3K4me1 in the absence of H3K4me3 selectively associates with distal regulatory regions [61]. H3K4me1 may function to protect genomic regions from repressors that bind unmodified H3K4 [62, 63] or alternatively as a docking site for factors that enhance the regulatory potential of the region by altering its accessibility [64]. This signature is found at enhancers that are active, poised or repressed and remains long after activity has seized [61, 65–67]. The presence of histone 3 lysine 27 acetylation (H3K27ac) is indicative of active promoters as well as enhancers [6, 65, 67–69]. The acetyltransferases p300 and CREB binding protein (CBP) that deposit H3K27ac are similarly used to identify enhancers [70]. Acetylated lysines direct regulatory activity by serving as docking sites for bromodomain containing cofactors [71]. However, they also influence chromatin compaction by attenuating histone-DNA interactions through the neutralization of electrostatic interactions [72]. Acetylation-based enhancer predictions are confirmed in reporter assays in ∼70 % of the cases presuming that a substantial fraction of the enhancer sequence is assayed [2, 23, 32, 35]. Furthermore, the discovery rate of enhancers using acetylated lysines is also relatively high [68]. Other assays to assess enhancer activity have been explored and were shown to be indicative of tissue-specific activity as well [73–75]. However, as CRE identification through the use of histone marks is relatively easy and robust, it currently remains the most frequently used method.

### Emerging Concepts from Large-Scale Identification of *cis*-Regulatory DNA

The epigenomic analysis of regulatory networks has substantially enhanced our understanding of how CREs operate, how these elements evolved across evolutionary time and according to which rules target genes are specified and controlled. For instance, the majority of distal regulatory elements were found to function as enhancers (over 400,000 predicted), often in a temporal and tissue-specific manner [1, 2, 54, 70]. In contrast, promoters, insulators and the overall topological structure of the genome were mostly found conserved between cell types [54, 76]. Similar observations were done over evolutionary time. While enhancer activity was shown to be poorly conserved across species, promoters and chromatin architecture were overall similar [77–81]. The fact that enhancers are highly tissue-specific as well as the fact that multiple enhancers can act together on a single gene in a redundant fashion, partially explains this lack of conservation [82, 83]. Furthermore, redundancy in recognition motifs allows enhancers to remain functionally conserved despite a lack of sequence similarity [51, 52, 84–86].

Different types of promoter and enhancer elements were discovered based on their activity. Enhancers can be active (bound by H3K27ac), while they can also exist in a poised state ready to be activated [65, 67]. A poised state is part of the transcriptional program that specifies cell state and gives the cell a set of transcriptional options to deploy rapidly in response to environmental cues. For instance, neurons are able to quickly integrate external stimuli and translate this into gene expression changes that can be either short or persist for longer periods. This signalling network, which is important during brain development to stabilize synapses, is also involved in synaptic plasticity and thus learning, cognition and memory [87]. Recent data in mouse cortical neurons, demonstrated that activity-dependent transcriptional changes are at least partially established through rapid epigenetic alterations in a pre-programmed poised enhancer network by the early response factor FOS [74, 88••].

While these principles represent some of the emerging concepts coming from large- scale CRE identification, one of the most important insights gained from these analyses is that much of the unexplained heritability of disease phenotypes might be located in deregulated noncoding regulatory regions [12].

### Misregulation of Enhancers in Disease

The importance of correct gene expression control is underscored by the misregulation or mutation of transcription factors in numerous diseases [4]. For instance, *ASCL1* mutations can give rise to Ondine’s curse, a severe neurological disorder that leads to fatal sleep apnoea [89]. However, the effects of mutations within transcription factors are often pleiotropic and thus affect multiple cell types resulting in severe developmental defects that are typically incompatible with life. Instead, many disorders are characterized by more subtle tissue-specific defects. For example, mutations in the coding sequence for sonic hedgehog (*SSH*) lead to early termination of embryonic development while a mutation within an enhancer that regulates *SSH* expression in the limb bud specifically causes preaxial polydactyly [9]. While this phenotype arises from faulty expression of *SSH*, misregulation is restricted to the developing limb and therefore irrelevant in other tissues.

Early studies targeting specific genomic loci by extensive long-range mapping have established a handful of enhancers as causative in very specific disorders such as the beta-globin enhancers in Thalassemia’s [90, 91] and a *RET* enhancer in Hirschsprung disease [92, 93]. After these observations, additional enhancer mutations were found in a variety of disorders including Pierre Robin syndrome [10], pancreatic agenesis [94] and congenital heart disease [95]. The latter underscored the modularity of enhancers as mutations in *TBX5* (T-box 5) result in congenital heart defects and limb malformations while mutations in single enhancers could decouple these phenotypes.

In addition, several different modes of enhancer deregulation were uncovered. In acute lymphoid leukaemia, aberrant transcriptional regulation was found to result from point mutations that created a new enhancer in front of the *TAL* (Transcription activator-like) oncogene [96]. Enhancer driven oncogene activation was also shown to occur as a result of enhancer translocations including the classic example of Burkitt lymphoma in which the *MYC* oncogene falls under the control of an immunoglobulin enhancer after a *t*(8;14) chromosomal translocation [97]. More recently, it was demonstrated that oncogene activation can occur after disruption of insulated chromatin neighbourhoods [98, 99]. As a result, expression of proto-oncogenes was increased through newly established long-range interactions. Finally, the epigenomic deregulation of enhancers by loss of DNA methylation was shown to be widespread in tumours [100]. These results demonstrate that diverse modes of enhancer misregulation can underlie a host of diseases and can explain the tissue-specific manifestation of such disorders.

### Enhancers as a Source for Common Variation in Disease Susceptibility

With more than 400,000 potential noncoding regulatory elements identified in the human genome, the mutational space for disease-causing events has increased substantially. The inability to explain disease heritability by gene mutations alone as well as the presence of more than 85 % of disease-associated variants in noncoding DNA [101] have strengthened the notion that much of the genetic variation that is relevant to disease lies within regulatory DNA (Fig. 1b). This has been supported by earlier extensive investigation of the *RET* gene locus for which a common variant within a *RET* enhancer was found to increase Hirschsprung disease susceptibility [102, 103]. Nevertheless, linking the disease-associated variant to specific regulatory elements often remained challenging. For instance, in depth analysis of a risk haplotype in the 5′ region of *SORL1* (sortilin-related receptor L) provided important new insight into the pathogenesis of Alzheimer’s disease while the exact polymorphism behind this effect remained elusive [104].

Based on these observations, the integration of genome-wide association studies with datasets of annotated enhancer elements has rapidly led to the discovery of potential disease-associated variants in predicted enhancer elements (Fig. 1b) [105–110]. This revealed that common disease variants preferentially occurred at enhancers in cell types known to be affected by the disease and therefore yielded a trove of candidates for further study. For example, while common variants that alter susceptibility to behavioural disorders preferentially occurred in foetal brain CREs [106], common variants associated with increased Parkinson’s disease (PD) susceptibility were found preferentially in CREs of the adult human brain (Fig. 1b) [111•]. In agreement with this, a number of neural transcription factor binding sites (e.g. Pax6 and Otx1) were disproportionally affected by variants associated with neuropsychiatric diseases and traits [106]. Several surprising observations were also made such as an unexpected link between B-cells and multiple sclerosis [106]. This suggested that specific cell types that are affected in disease could be deducted from the integration of cell type-specific enhancers and genome-wide association (GWAS) data (Fig. 1b). Similarly, in a more recent study, common variation in Alzheimer’s disease was linked to the immune system suggesting that much of the regulatory variation underlying this disease may not be intrinsic to neurons [112]. This underscores the relevance of integrating the two data types to uncover new cell types that are involved in disease susceptibility but also to prioritize the regulatory elements that are likely affected by genomic variation. Furthermore, it solidifies the notion that intra-individual genetic variation, which is most pronounced at regulatory DNA [113, 114], plays an integral role in determining disease susceptibility [108–110].

### Parkinson’s Disease-Associated Variation Within Human Brain Enhancers

While genomic variation within CREs is likely to have functional consequences, the link between potentially relevant enhancer variants and disease has to be experimentally verified. This has been done for a handful of disorders such as the *FTO* (fat mass and obesity-associated) locus in obesity [11], *LMO1* (LIM domain only 1) in neuroblastoma predisposition [115] and *BCL11A* (B-cell lymphoma/leukaemia 11A) in sickle cell anaemia [116•]. However, proper validation is difficult, especially for complex diseases such as neurodegenerative and neuropsychiatric disorders in which the combined activity of several regulatory elements on multiple genes may underlie pathophysiology. Furthermore, given the moderate effect size of common variations on disease susceptibility, the effect size on gene expression may also be modest.

We have previously linked several genetic variants associated with altered PD susceptibility to enhancers of the adult brain [111•]. These included CREs in important PARK loci such as the PARK16, PARK17 and the PARK8 loci, containing *LRRK2* (Leucine-rich repeat kinase 2), a gene that is mutated in autosomal dominant PD [117]. Similarly, we identified an intronic enhancer in the *SNCA* (α-synuclein) gene that contained two genomic variants rs356168 and rs3756054 [111•] that were in perfect linkage with earlier described PD risk alleles rs2736990 and rs11931074. These were initially discovered in cohorts of European and of Asian descent, respectively [118, 119]. This suggested that the different common variants in two populations converged at one enhancer element. In support of a role for enhancer variation, we found that both of these linked variants altered transcription factor binding sites in the predicted enhancer. Furthermore, the regulatory region acted as an enhancer in transgenic mouse assays, phenocopying the expression of *SNCA* in E11 mouse embryos. Finally, the enhancer was shown to directly target the *SNCA* promoter in chromosome conformation capture experiments in human brain tissue [111•]. These data firmly established the newly identified CRE as a bona fide *SNCA* enhancer.

As enhancers are mostly cell type- and context-specific, a correct model system and environment needs to be established to explore the functional consequences of enhancer alteration. This can be particularly difficult for the nervous system [120]. In addition, the effect size of enhancer variation is likely modest, given the fact that a 50 % increase in *SNCA* expression will cause PD [121]. A recent study dealt with all of these issues by employing an elegant experimental set up to analyse the influence of genetic variation within the intronic *SNCA* enhancer using allele-specific *SNCA* expression analysis in embryonic stem cell-derived neurons [122••]. Slight, but consistent increased expression of *SNCA* was observed for the Parkinson’s variant of rs356168 but not for variant rs3756054. This suggests that the latter SNP may not contribute to disease susceptibility and that other variants with lower linkage may still have to be explored. Nevertheless, these data did confirm our previously proposed link between the rs356168 enhancer variant and PD susceptibility. This underscores the importance of rigorous validation of enhancer variants within CREs and stands as a testimony for the huge task ahead.

## Conclusion

CREs play a pivotal role in the proper establishment of the gene expression programs that determine cell state. Following a decade of epigenomic exploration to chart CREs in the human genome, we are now starting to unravel some of the regulatory networks that contribute to a host of brain disorders as well as to individual variation in disease susceptibility. Since the start of the ENCODE project, to the more recent report of 111 epigenomes by the Roadmap Epigenetics Consortium, both consortia have added hundreds of datasets of different epigenetic footprints in a host of human tissues including 8 adult and 2 foetal brain samples [6]. However, to capture the full complexity of the brain many more anatomical regions still need to be explored. Our analysis of 87 anatomically distinct regions in the human brain was a confirmation of this, as many specialized structures within the brain, that were not included in other analyses, contributed significantly to the total repertoire of predicted CREs in the human brain [111•]. Follow-up analyses to identify regulatory changes that could be relevant to brain diseases are underway with consortia such as PsychENCODE focussing on neuropsychiatric diseases including autism and schizophrenia [123]. These studies will have to be balanced between the number of epigenetic footprints analysed, the number of patients included and the number of anatomical regions required. Furthermore, new (single cell) methods will have to be optimized to tackle the investigation of rare cell types within brain tissue samples.

While many potential links between neurodegenerative disease and regulatory changes have already been revealed, extensive validation experiments are required to confirm these in detail. This is challenging because of the modest effect size of sequence variation on enhancer function and the potential involvement of multiple genes as well as the requirement of relevant model systems in which activity can be properly measured. CRISPR-Cas9 mediated engineering of human embryonic stem cells will prove a powerful tool to analyse the effect of enhancers on their cognate target gene [124], especially since these cells can be used to generate a host of different cell types in the brain [120]. However, as multiple cell types can be involved in complex diseases, the implementation of more complex culture systems such as organoid cultures, that mimic cortical development, may be of use [125, 126]. Finally, the current focus on common variation will have to be complemented with research on structural and rare variants, as those are also likely to affect enhancer activity [105]. Since transcription factor binding sites are often degenerate, allowing multiple variations to activate or inactivate enhancer elements, disease-causing mutations will likely be rare. Therefore, genetic as well as epigenetic screens to reveal rare variants and to assay their consequences on enhancer activity will need to be explored.
